# NOX1-induced accumulation of reactive oxygen species in abdominal fat-derived mesenchymal stromal cells impinges on long-term proliferation

**DOI:** 10.1038/cddis.2015.84

**Published:** 2015-04-16

**Authors:** M Sela, G Tirza, O Ravid, I Volovitz, I Solodeev, O Friedman, D Zipori, E Gur, Y Krelin, N Shani

**Affiliations:** 1 The Plastic Surgery Department Tel Aviv Sourasky Medical Center, Tel Aviv, Israel; 2 The Department of Molecular Cell Biology, Weizmann Institute of Science, Rehovot, Israel; 3 The Neurosurgery Department, Tel Aviv Sourasky Medical Center, Tel Aviv, Israel

## Abstract

Mesenchymal stromal cells (MSCs) are multipotent and can be derived from different adult tissues including fat. Our repeated attempts to produce long-term proliferative cultures of rat abdominal adipose stem cells (aASCs) under normal oxygen concentration (21%) were unsuccessful. We set to examine the events controlling this cytostasis of aASCs and found that it resulted from overproduction of reactive oxygen species (ROS) that led to apoptosis. ROS overproduction in aASCs was accompanied by increased expression of NOX1 but not of NOX2 or NOX4. NOX family members are an important source of intracellular ROS pointing to NOX1 involvement in ROS accumulation. This was verified when aASCs that were grown under 3% oxygen conditions expanded long term, displaying reduced NOX1 expression and decreased ROS accumulation. NOX1 involvement in aASC cytostasis was reaffirmed when cells that were expanded under normoxic conditions in the presence of a specific NOX1 inhibitor, ML171, demonstrated reduced ROS accumulation, reduced apoptosis and long-term expansion. aASC expansion arrest was accompanied also by a weak fat differentiation and migratory potential, which was enhanced by NOX1 inhibition. This suggests an inhibitory role for NOX1-induced ROS overproduction on aASCs, their fat differentiation and migratory potential. In contrast to aASCs, similar cells produced from subcutaneous fat were easily expanded in normoxic cultures, exhibiting low ROS concentrations, a low number of apoptotic cells and improved fat differentiation and migration. Taken together, our results show, for the first time, that NOX1-induced ROS accumulation halts ASC expansion and reduces their differentiation and migratory potential under normoxic conditions. Importantly, this phenotype comprises a tissue-specific signature as it was evident in aASCs but not in subcutaneous ASCs. NOX-induced ROS accumulation and cytokine production by fat are part of the metabolic syndrome. The similarity of this phenomenon to aASC phenotype may indicate that they arise from similar molecular mechanisms.

Mesenchymal stromal cells (MSCs) are multipotent progenitor cells that are produced and propagated from a wide range of adult tissues.^1^ MSCs were suggested to originate from a perivascular source in various adult tissues.^2^ Although they were originally derived mainly from bone marrow, adipose-derived MSCs (ASCs) were recently demonstrated to harbor properties similar to bone marrow-derived MSCs.^3^ MSCs that were expanded under ‘hypoxic' conditions (1–5% oxygen) demonstrated improved culture expansion, differentiation and genomic stability compared with MSCs that were grown under ‘normoxic' conditions (atmospheric oxygen level).^4, 5, 6^ Reduced reactive oxygen species (ROS) accumulation was suggested as a possible explanation to the improved expansion of MSCs under low oxygen conditions.^7^

ROS are produced in cells mostly by the mitochondrial oxidative phosphorylation process or as cellular signaling molecules mainly by the family of NOX NADPH oxidases.^8^ NOX family members generate superoxides and other downstream ROS products.^8, 9^ NOX1 the first NOX2 homolog to be described^10, 11^ is highly expressed in colon epithelium, and expressed in many other tissues and cells, including fibroblasts.^9, 12, 13^ ROS overproduction leads to many destructive cellular processes, such as aging, DNA damage and apoptosis.^14, 15^ Importantly, NOX-induced ROS accumulation in fat tissue during obesity was shown to be the cause of the deregulated production of adipocytokines and the induction of the metabolic syndrome.^16, 17, 18^ Development of the metabolic syndrome was correlated with the accumulation of abdominal/visceral fat rather than the accumulation of total body fat, indicating the importance of abdominal fat in the development of this syndrome.^19^

Here we show that the inability of abdominal rat ASCs to reach long-term culture expansion (i.e expansion arrest), their weak fat differentiation and migratory potential and their increased cytokine expression results from NOX1-induced ROS accumulation that leads to their apoptotic death. Specific inhibition of NOX1 enabled long-term propagation of abdominal ASCs (aASCs) and their improved fat differentiation and migration. The role of the tissue origin in the aASC phenotype was demonstrated as ASCs from subcutaneous fat displayed reduced ROS accumulation, long-term culture propagation, improved fat differentiation and reduced cytokine expression compared with aASCs.

## Results

### Abdominal adipose-derived rat MSCs expansion arrest in early passages involves apoptotic cell death

**Figure 1:**
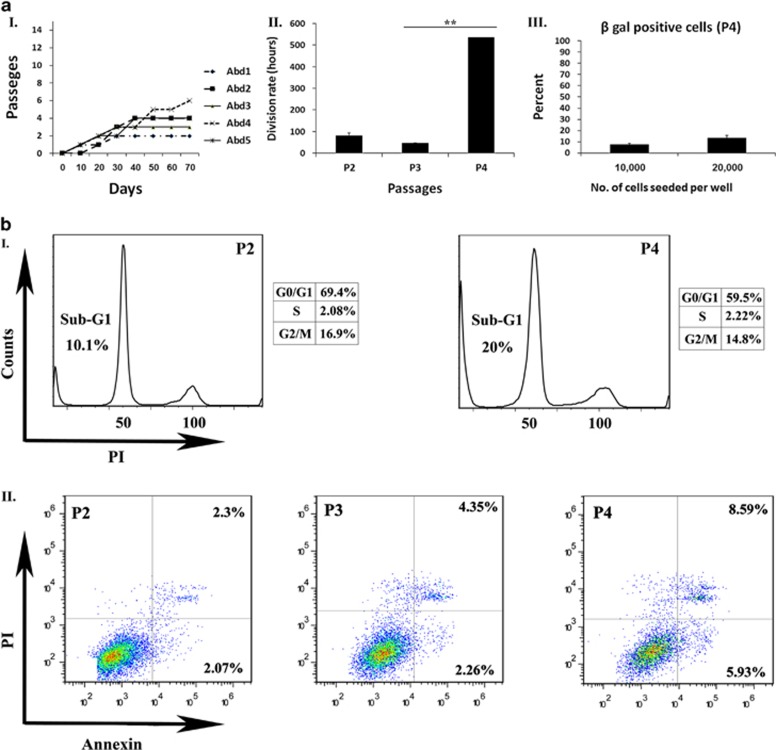
Abdominal ASCs undergo an expansion arrest at early passages that is promoted by apoptotic cell death. (**a**I) Cells were isolated from intra-abdominal fat and examined for their ability to undergo long-term expansion in culture (five independent repeats were performed). All cells underwent a propagation arrest during passages 2–6, in which they stopped replicating and were unable to reach a confluent state. (**a**II) Cells at passages 2–4 were seeded at a density of 2 × 10^4^ cells per 6 cm plate. The division rate was assessed by calculating the logarithmic growing phase. (**a**III) Cells at passage 4 were seeded at a density of 1 × 10^4^ or 2 × 10^4^ cells per 6 cm plate. Gal activity was detected by staining with *β*-gal reaction buffer. Senescent cells (dyed blue) were quantified by light microscopy. Results are presented as the percent of total dyed cells per a high power field. (**b**I) Cell cycles in increasing passages were evaluated by PI staining. (**b**II) The percentage of apoptotic cells was evaluated by the PI/Annexin staining and analyzed by FACS (fluorescence-activated cell sorting). Error bars represent S.D. (Student's two-tailed *t*-test for equal variance). **P<0.01 and *P<0.05

The most abundant source of fat tissue in rodents is the inguinal pads located within their abdominal cavity. Inguinal fat pad resembles abdominal/visceral human fat.^20, 21^ Repeated attempts (*N*>5) to propagate rat ASCs (characterization of abdominal ASCs is shown in Supplementary Figure 1) from an abdominal source using both regular culture conditions (Dulbecco's modified Eagle's medium (DMEM)+10% FCS) and commercial mediums all resulted in an expansion arrest during passages 2–6, in which the cells were unable to reach a confluent state (Figure 1aI and data not shown) and displayed a marked increase in their population doubling time (Figure 1aII). Our evaluation of aASCs during culture did not demonstrate a significant increase in the percent of senescent cells with subsequent passages (senescent cells reached a maximal of ~10% of total cells as demonstrated by *β*-gal-positive cells (Figure 1aIII). Similarly, cell-cycle analysis in increasing passages did not exhibit any significant cell-cycle arrest but rather an increase of the sub-G1 population with passage, indicating apoptotic cell death (Figure 1bI). The apoptotic fate of aASCs was further confirmed by the demonstration of an increased proportion of Annexin-positive apoptotic cells correlating with passage progression and the observed expansion arrest (Figure 1bII). It therefore appears that the expansion arrest of aASCs can be attributed largely to the induction of apoptosis.

### Abdominal ASC expansion arrest is accompanied by increased expression of NOX1, antioxidant genes and cytokines

**Figure 2:**
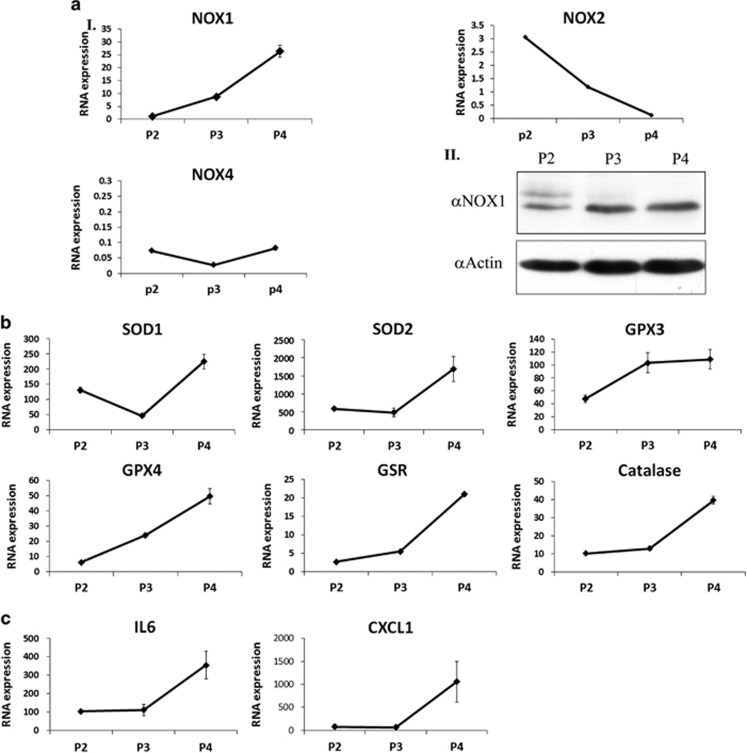
Abdominal ASC expansion arrest is accompanied by increased expression of NOX1, antioxidant and cytokines genes. The expression of NOX2 and NOX4 was examined by quantitative reverse transcription-PCR (qRT-PCR) (**a**I). The expression of NOX1 was examined at both the RNA level by qRT-PCR and the protein level by western blot (**a**I and **a**II, respectively). (**b** and **c**) The RNA expression levels of different antioxidant enzymes and various cytokines were evaluated by qRT-PCR at the indicated passages. Error bars represent S.D. Results were repeated three times following the same trend and are presented in Figures 3 and 4

Given the apoptotic fate of aASCs, we assumed that these cells may undergo an oxidative stress response similar to the one experienced by abdominal fat during obesity. Evaluation of the mRNA expression levels of the NADPH oxidases, NOX1/2/4, in cultured aASCs in passages 2–4 revealed a marked increase in NOX1 expression (a 23-fold increase) but not of NOX2 and 4 (Figure 2aI). The increase in NOX1 mRNA and protein expression (Figures 2aI and II, respectively) in correlation with the induction of apoptosis and expansion arrest may point to a NOX1-induced oxidative stress in aASCs. An increased gene expression of some of the major antioxidant enzymes (i.e. superoxide dismutase (SOD) 1 and 2, glutathione peroxidase (GPX) 3 and 4, catalase and glutathione-disulfide reductase (GSR) in conjunction with the upregulated NOX1 expression, further supports the presence of an oxidative stress in aASCs (Figure 2b). aASC cultures also exhibited a passage-dependent increased expression of IL-6 and CXCL-1, both inducing inflammation (Figure 2c). Confirmation of culture purity during ASC passaging (Supplementary Figure 1) eliminated the possibility of cytokine secretion by culture-contaminating hematopoietic cells.

### Culturing aASCs under 3% oxygen (hypoxia) reduces ROS levels, NOX1 and cytokine expression and protects aASCs from expansion arrest

**Figure 3:**
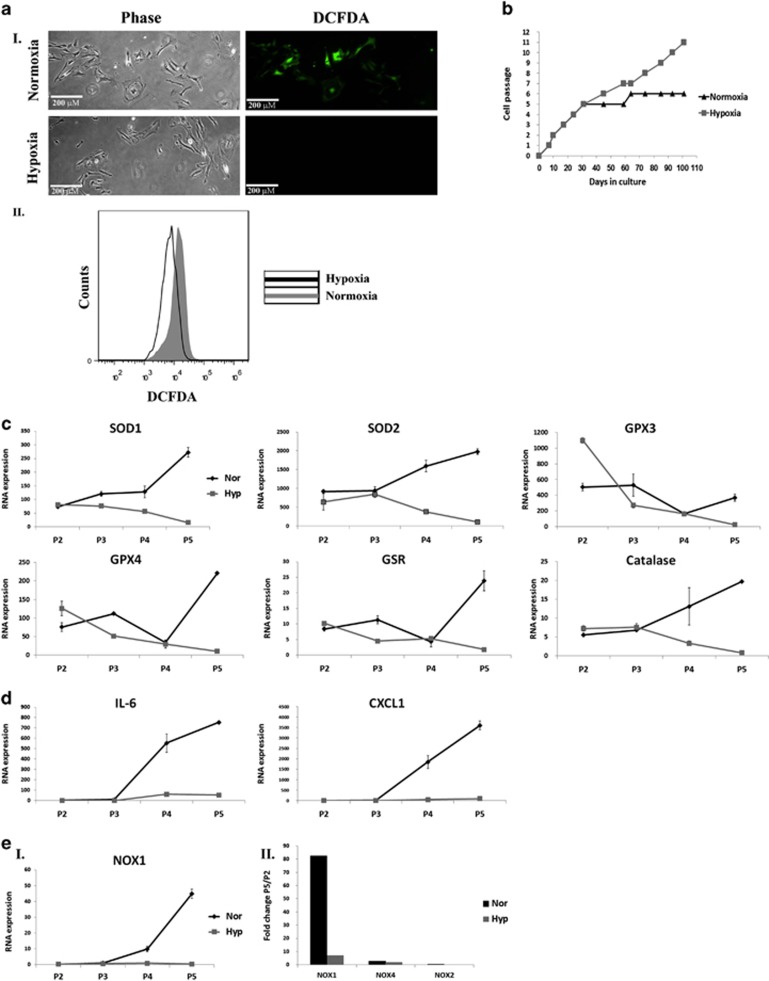
Hypoxic conditions (3% oxygen) inhibit ROS accumulation by abdominal ASCs and allow their long-term propagation. Abdominal fat was isolated and ASCs were propagated under ‘normoxic' conditions (21% oxygen) or hypoxic conditions (3% oxygen). The following analyses were performed on aASCs that were grown under different oxygen conditions. (**a**) ROS accumulation was detected by DCFDA staining and analyzed by fluorescence microscopy or by FACS (fluorescence-activated cell sorting) (**a**I and **a**II, respectively). (**b**) Growth curves of aASCs cultured at 3% oxygen (gray line) and 21% oxygen (black line). Only cells that were cultured at 3% oxygen were able to undergo long-term expansion. (**c** and **d**) RNA expression of various genes was compared by quantitative reverse transcription-PCR (qRT-PCR) analysis between cells that were grown under hypoxic conditions (gray line) to those grown under normoxic conditions (black line) at the indicated passages. (**e**) The expression of NOX1, NOX2 and NOX4 were evaluated in cells that were grown under hypoxic (gray) or normoxic (black) conditions by qRT-PCR. The expression is presented as RNA expression (**e**I) or as the fold of expression between passage 5 and 2 (**e**II). Error bars represent S.D. Results were repeated two times following the same trend

Culturing human MSCs under low oxygen conditions was previously demonstrated to reduce ROS production by inducing a shift from oxidative phosphorylation to glycolysis.^7^ Culturing aASCs under 3% oxygen reduced ROS accumulation compared with aASCs that were grown under normoxic (21%) conditions (Figure 3aI and II) and salvaged them from their expansion arrest (Figure 3b). Reduction in ROS accumulation in aASCs that were cultured under 3% oxygen was also accompanied by a reduced gene expression of antioxidant enzymes (Figure 3c) and inflammatory cytokines (Figure 3d) compared with aASCs cultured under normoxic conditions. Importantly, hypoxic culture conditions markedly inhibited the increase in NOX1 compared with normoxic conditions (Figures 3eI and II). No significant increase in NOX2 and 4 expression was seen under both normoxic and hypoxic conditions (Figure 3eII). These findings further support NOX1-dependent ROS accumulation as the main cause of the expansion arrest of aASCs.

### Inhibition of NOX1 activity by a specific inhibitor, reduces ROS accumulation, apoptotic death and expansion arrest of aASCs cultured under normoxic conditions

**Figure 4:**
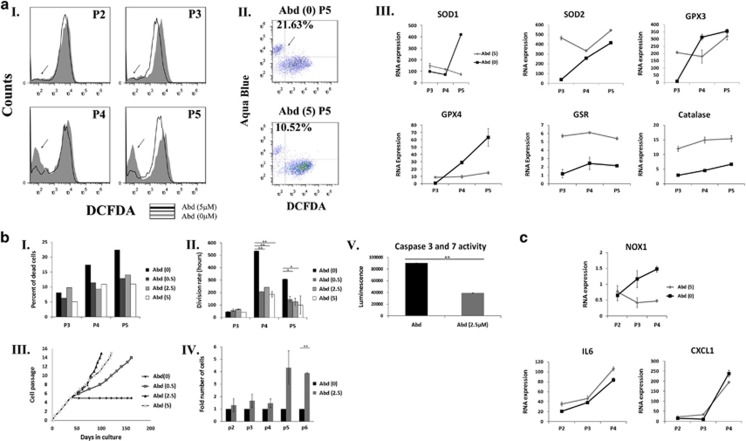
A specific NOX1 inhibitor reduces ROS accumulation and promotes long-term expansion of aASCs. Abdominal ASCs (Abd) were propagated under normoxic oxygen conditions (21% oxygen) in the presence of an NOX1-specific inhibitor ML171 (0, 0.5, 2.5 and 5 *μ*M). The following analyses were performed at the indicated passages (P). (**a**I) ROS accumulation was evaluated by FACS (fluorescence-activated cell sorting) analysis using DCFDA staining, and the results of untreated aASCs compared with aASCs treated with 5 *μ*M inhibitor are shown. (**a**II) Starting from P3, an additional population with a low DCFDA signal appeared (marked by an arrow). Dual staining of cells by DCFDA and aqua blue dye (an amine viability dye) was performed and analyzed by FACS. Results demonstrated that the majority of low DCFDA cells were aqua blue-positive dead cells. (**a**III) RNA expression level of different antioxidant enzymes was compared between cells that were cultured with NOX1 inhibitor (gray line) or without it (black line). Error bars represent S.D. (**b**I) The percent of dead cells was evaluated by aqua blue staining. The division rate of untreated aASCs was compared with aASCs that were treated with different concentrations of NOX1 inhibitor (**b**II), and their long-term propagation of cells was evaluated (**b**III). (**b**IV) The number of cells of untreated aASCs and aASCs treated with 2.5 *μ*M NOX1 inhibitor was compared at the indicated passages in two independent experiments. (**b**V) Activities of caspase-3/7 in untreated aASCs and aASCs treated with 2.5 *μ*M were measured by a Caspase-Glo 3/7 Assay Kit. Error bars represent S.E.M. (Student's two-tailed *t*-test for equal (**b**II) or unequal (**b**IV) variance). **P<0.01 and *P<0.05. (**c**) The RNA expression level of NOX1 and various cytokines was compared between untreated aASCs and aASCs treated with 5 *μ*M NOX1 inhibitor by quantitative reverse transcription-PCR (qRT-PCR) at the indicated passages. Error bars represent S.D. Untreated aASCs (Abd (0)). aASCs treated with 0.5 *μ*M, 2.5 *μ*M and 5 *μ*M NOX1 inhibitor (Abd (0.5), Abd (2.5), Abd (5), respectively)

To substantiate a relation between NOX1 activity and the aASC cytostatic phenomenon, aASCs were cultured under normoxic conditions with or without a specific NOX1 inhibitor, ML171,^22^ in various concentrations starting at passage 2. ML171-treated aASCs displayed a significant ROS reduction (as noted by reduced 2′,7-dichlorodihydrofluorescein diacetate (DCFDA) staining) 1 week after the initiation of treatment (passage 2) (Figure 4aI). This trend was evident in all inhibitor concentrations and in all the passages examined until passage 5, when untreated aASCs stopped propagating (Figure 4aI and Supplementary Figure 2). Interestingly, an additional population with a low DCFDA signal appeared in untreated aASCs and, to a lesser extent, in aASCs treated with NOX1 inhibitor at passage 3 (Figure 4aI and Supplementary Figure 2). Untreated aASCs had already been demonstrated to exhibit significant apoptosis that increased during passaging (Figure 1b). Double-staining of the cells with DCFDA and aqua blue (an amine viability dye) demonstrated that the majority of cells that were low in DCFDA staining were aqua blue-positive dead cells (Figure 4aII). NOX1 inhibition was able to reduce the proportion of DCFDA low aqua blue-positive dead cells (Figure 4aII). As shown above (Figures 2b and 3b), ROS accumulation in untreated aASCs was accompanied by an increase in the gene expression of antioxidant genes. This was partially repeated in treated aASCs, with the most prominent changes being a reduced expression of SOD1 and GPX4 and an increased constant expression of catalase and GSR from as early as passage 3 (Figure 4aIII). NOX1 inhibition also resulted in a ~40% reduction in the number of dead cells at passages 4 and 5 compared with untreated cells in all inhibitor concentrations (Figure 4bI and Supplementary Figure 3). In accordance with the reduced death in treated aASCs, NOX1 inhibitor reduced the doubling time of treated cells at all concentrations, compared with untreated cells (Figure 4bII). NOX1 inhibition also salvaged cells from the expansion arrest that was observed in untreated cells at passage 5 and preserved long-term expansion capability of aASCs (the rate of expansion was concentration dependent) (Figure 4bIII). The increased proliferation of treated cells was also evident from their higher numbers in passages 5 and 6 compared with the untreated controls (Figure 4bIV). NOX1-induced cytostasis was accompanied by caspase-3 and -7 activation, which was inhibited in treated cells indicating the involvement of ROS-dependent caspase activation in aASC death (Figure 4bV and Supplementary Figure 4c). Although NOX1 inhibition was able to reduce cell death induction in aASCs, the inhibition was not absolute. Consequently, NOX1 inhibition reduced NOX1 expression in the treated aASCs, but was unable to prevent the increase in inflammatory cytokine expression in correlation with passage progression (Figure 4c). It is concluded that NOX1 inhibition in aASCs resulted in reduced ROS accumulation, caspase activation and cell death and inhibited their expansion arrest.

### Cultured subcutaneous ASCs do not undergo oxidative stress-induced expansion arrest

**Figure 5:**
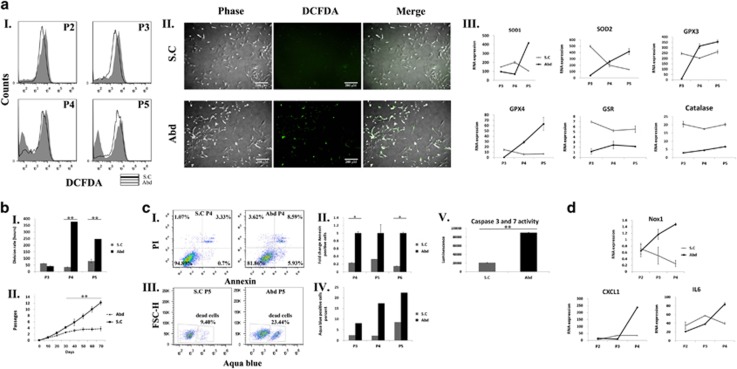
scASCs display reduced NOX1 expression, reduced ROS accumulation and long-term expansion. aASCs (Abd) and scASCs (SC) were cultured under normoxic conditions (21% oxygen) and the following analyses were performed at the indicated passages (P). (**a**) ROS accumulation was detected by DCFDA staining and analyzed by FACS (fluorescence-activated cell sorting) (**a**I) and by fluorescence microscopy (**a**II). (**a**III) The mRNA expression level of different antioxidant enzymes and various cytokines was evaluated by quantitative reverse transcription-PCR (qRT-PCR) at the indicated passages. Error bars represent S.D. (**b**I) Cells at passages 3–5 were seeded at a density of 2 × 10^4^ cells per 6 cm plate. The division rate was assessed by calculating the logarithmic growing phase. (**b**II) Growth curves of ASCs from an abdominal fat source (dotted lines) and from subcutaneous fat (continuous line). Only scASCs cells were able to undergo long-term expansion. Experiments were performed on five independent preparations from each fat source. Error bars represent S.D. **P<0.01 and *P<0.05 (Student's two-tailed *t*-test for equal variance). (**c**I) A percentage of apoptotic cells from aASCs and scASCs was evaluated by PI/Annexin staining and analyzed by FACS. (**c**II) The results represent the fold change of Annexin-positive cells from aASCs (black bars) and scASCs (gray bars). Experiments were carried out in two independent ASC preparations. Error bars represent S.E. **P<0.01 and *P<0.05 (Student's two-tailed *t*-test for unequal variance). (**c**III and **c**IV) The percent of dead cells was evaluated by aqua blue staining at the indicated passages. (**c**V) Activities of caspase-3/7 in aASCs and scASCs were evaluated by a Caspase-Glo 3/7 Assay Kit. (**d**) RNA expression level of NOX1 and various cytokines was evaluated by qRT-PCR at the indicated passages. Error bars represent S.D. of subcutaneous ASCs (S.C) and abdominal ASCs (Abd). Untreated aASCs were cultured in parallel to both scASCs and to aASCs that were treated with an NOX1 inhibitor and are therefore used as control in both Figures 4 and 5

To evaluate whether the cytostatic phenotype that was demonstrated by aASCs was shared by ASCs produced from other fat sources, subcutaneous fat was used. Cultured subcutaneous ASCs (scASCs) exhibited a reduced ROS accumulation compared with aASCs (Figures 5aI and II). Similar to inhibitor-treated aASCs, scASCs revealed lower expression levels of SOD1 and GPX4 and a higher constant expression levels of catalase and GSR compared with aASCs starting from passage 3 (Figures 4aIII and 5aIII). This indicates similar downstream mechanisms that lead to reduced ROS accumulation in both aASC-treated cells and scASCs. Reduced ROS accumulation in scASCs was also accompanied by a reduced doubling time (Figure 5bI), by the ability to perform long-term expansion in culture (Figure 5bII) and by a large reduction in apoptotic cell death compared with aASCs and by reduced caspase activation (Figure 5c). The difference in phenotypes between scASCs and aASCs was also evident by the reduced gene expression of NOX1 and of inflammatory mediators (Figure 5d). Thus, aASCs possess a tissue-specific signature that is not found in scASCs.

### aASCs demonstrate a weak fat differentiation potential compared with scASCs

**Figure 6:**
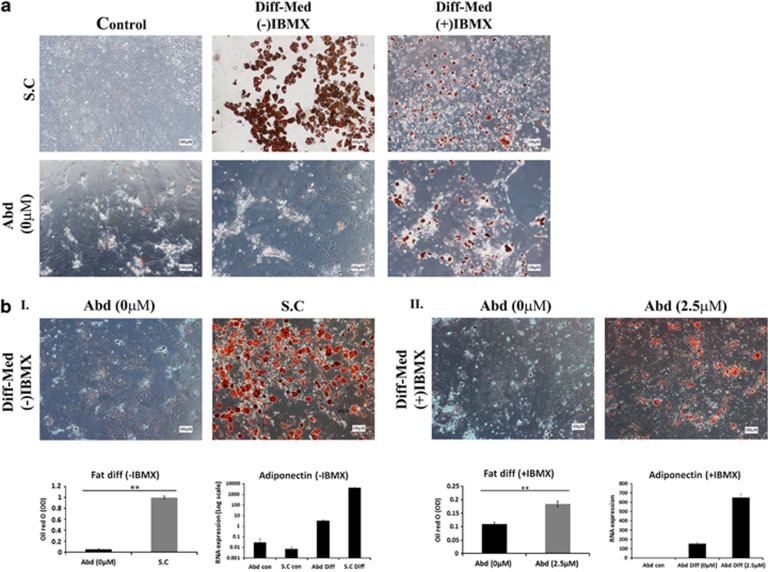
Inhibition of NOX1 activity improves fat differentiation capacity of aASCs. (**a**) aASCs (Abd (0)) and scASCs (S.C) were cultured under normoxic conditions (21% oxygen) with or without induction media for 3 weeks to induce fat cell differentiation with or without IBMX. Differentiation into fat was detected by Oil Red O staining (representative images are presented). The aASCs are seen to demonstrate a weak fat differentiation potential compared with scASCs. (**b**) The fat differentiation capacity of scASCs (I) or of aASCs that were treated with an NOX1 inhibitor (II) was compared with untreated ASCs under normoxic oxygen conditions. Differentiation was induced by a differentiation medium with IBMX. Following the differentiation cells were stained by Oil Red O, photographed and the stain was extracted and quantified. Alternatively, RNA was extracted from differentiated cells and the expression level of adiponectin was evaluated by quantitative reverse transcription-PCR (qRT-PCR). Inhibition of NOX1 activity improved the fat differentiation capacity of aASCs. Error bars represent S.E. **P<0.01 and *P<0.05 (Student's two-tailed *t*-test for equal variance)

Fat differentiation of ASCs was induced by using a basic fat differentiation medium with or without IBMX (3-isobutyl-1-methylxanthine). Differentiation was observed in scASCs by both media, although the differentiation was more robust in medium without IBMX (Figure 6a). In contrast to scASCs, differentiation of aASCs was achieved only in medium containing IBMX and not in IBMX (−) medium and was weaker than that of scASCs, even in the presence of IBMX. The increased fat differentiation potential of scASCs was further substantiated by their increased Oil Red O signal following dye extraction and by their increased adiponectin RNA expression compared with aASCs (Figure 6bI). Thus, aASC and scASC phenotypes differed not only in their propagation potential but also in their fat differentiation potential.

### Inhibition of NOX1 activity improves the fat differentiation capacity of aASCs

NOX1 inhibition in aASCs was unable to promote fat differentiation in cells that were cultured in an IBMX (−) differentiation medium (data not shown). In contrast, NOX1 inhibition in aASCs that were cultured in IBMX (+) differentiation medium led to their improved fat differentiation compared with untreated aASCs (Figure 6bII). It would therefore appear that NOX1-induced ROS accumulation attenuates the differentiation capacity of aASCs.

### aASCs demonstrate a weak migration potential compared with scASCs that is improved by NOX1 inhibition

**Figure 7:**
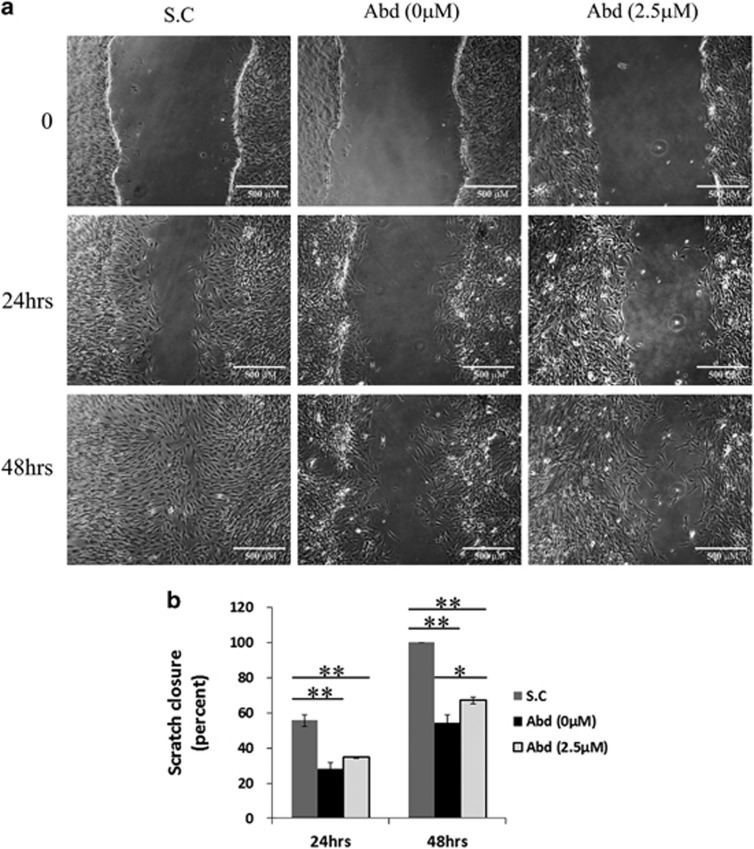
aASCs demonstrate a weak migration potential compared with scASCs that is improved by NOX1 inhibition. scASCs and aASCs either treated with a specific NOX1 inhibitor or untreated were cultured to a confluence state and three parallel scratches were made on the cell monolayer. Cells were incubated for 48 h in complete medium. Scratch closure was photographed immediately, 24 h and 48 h after scratch (**a**). The extent of healing was defined by measuring the wounded area and reported as the percentage of scratch closure (**b**). Error bars represent S.E. **P<0.01 and *P<0.05 (Anova)

One of the inherent traits of all mesenchymal cells, which is evident already in embryonic development, is their migratory capabilities.^23^ Comparison of aASCs and scASCs by scratch assay revealed significantly superior migratory properties of scASCs (Figures 7a and b). The reduced migratory potential of aASCs can be partially explained by their NOX1-induced ROS accumulation since specific inhibition of NOX1 significantly increased their migration abilities compared with untreated control 48 h following scratch creation (Figures 7a and b).

## Discussion

ROS production has been recognized over the past decade as being involved in many cellular processes. ROS overproduction, however, may result in damage to tissues and cells that translates into cell death and aging. We found that aASCs constantly failed to produce long-term cultures under various culture conditions, and showed a weak fat differentiation and migratory potential. We demonstrate here, for the first time, that the expansion arrest of aASCs resulted from NOX1-induced ROS accumulation during culture, and that their consequent apoptotic death, was accompanied by proinflammatory cytokine expression. In contrast to aASCs, ASCs that were derived from subcutaneous tissue were able to propagate long term in culture because of their reduced ROS accumulation and reduced cell death. Moreover, they did not demonstrate increased cytokine expression, and showed strong fat differentiation and a strong migratory potential. It would therefore appear that the phenotype of aASCs is controlled by a tissue-specific signature that originates from their tissue source. Importantly, NOX-induced ROS accumulation and the development of a proinflammatory phenotype are known to occur in abdominal fat during obesity, leading to the development of the metabolic syndrome. The similarity between the aASCs phenotype in culture and abdominal fat during obesity may suggest that both processes originate from similar molecular mechanisms.

### ROS accumulation is responsible for aASC expansion arrest

We found that aASCs that are expanded under normoxic conditions are unable to perform long-term culture because of ROS over accumulation, which leads to increased apoptosis. Similar to previous reports,^7^ aASCs that were grown in low oxygen conditions (3%) demonstrated reduced ROS accumulation and underwent long-term expansion (until passage 15). This substantiates ROS accumulation as a major cause for aASC expansion arrest. Bone marrow MSCs grown in normoxic conditions had been shown to undergo apoptosis induced expansion arrest in a p53-dependent manner by mitochondrial ROS overproduction.^4^ We were unable to find a direct involvement of p53 in aASCs expansion arrest (data not shown). Interestingly, expansion arrest of aASC was accompanied by increased expression of IL-6 and CXCL-1, which were previously suggested to be involved in oncogene-induced senescence,^24, 25^ but only by ~10% of β-gal-positive cells. Thus, despite the low percent of *β*-gal-positive cells, aASC expansion arrest may involve both apoptosis and senescence. Human fibroblasts reportedly responded to H_2_O_2_ by undergoing senescence, apoptosis or both in a concentration-dependent manner.^26^ It is thus feasible that aASC expansion arrest is controlled by both senescence and apoptosis that occur during to the gradual accumulation of ROS.

### NOX1 activity is responsible for ROS accumulation in aASCs

ROS accumulation in aASCs was accompanied by increased expression of NOX1 but not of NOX2 and NOX4, pointing to NOX1 as the source of ROS overproduction. Culture of aASCs under 3% oxygen resulted in reduced expression of NOX1 compared with aASCs that were grown under normoxic conditions, further indicating exaggerated activity of NOX1 as a probable cause for ROS accumulation in aASCs. Physiological oxygen levels in fat tissue were suggested as being in the range of 3–10%.^27^ In this manner, normoxic culture conditions supposedly expose ASCs to hyperoxia. Exposure of endothelial lung cells to hyperoxia was demonstrated to induce ROS accumulation first in a mitochondrial-dependent manner and then in an NOX1-dependent manner.^28, 29^ The increased expression of NOX1 solely under normoxic conditions and not under 3% oxygen (representing physiological conditions) suggests that the NOX1-induced ROS accumulation in aASCs occurs through a similar chain of events.

To further assess NOX1 involvement in aASC ROS accumulation, we chose to propagate aASCs in the presence of a specific NOX1 inhibitor termed ML171.^22, 30^ To ensure the inhibitor specifically targeted NOX1, we used it in three concentrations (0.5, 2.5 and 5 *μ*M), all of which significantly lower than 10 *μ*M in which residual activity of the inhibitor was also observed on other NOX family members.^22^ Reduced ROS accumulation and cell death and long-term expansion was observed in aASCs that were treated with all 3 concentrations of the inhibitor compared to untreated cells, thereby providing a strong correlation between NOX1 activity and ROS accumulation. Interestingly NOX1 inhibition in aASCs that were cultured under hypoxic conditions did not reduce ROS accumulation in the cells further supporting the reduced NOX1 activity under hypoxic conditions (Supplementary Figure 4A). We further found that aASC apoptosis occurred through caspase-3 and -7 activation, which was suppressed by NOX1 inhibition. This further supports NOX1 activity as an initiator of aASC apoptotic fate.

### NOX-induced ROS accumulation in abdominal ASCs demonstrates a tissue-specific phenotype that may be related to the metabolic syndrome

NOX-induced ROS accumulation in white adipose tissue during obesity is known to promote inflammation and cytokine production as well as to promote the development of the metabolic syndrome.^16, 17, 18^ Importantly, metabolic syndrome development is presumably affected mainly by the accumulation of abdominal/visceral fat and not by the accumulation of total fat, indicating a major role for abdominal/visceral fat in obesity-related morbidity.^19^ The partial resemblance in the phenotypes demonstrated by abdominal fat during obesity and aASCs led us to speculate that aASCs bear a tissue-specific signature that will not be present in ASCs that are produced from other adipose sources. This notion was confirmed by five independent preparations of subcutaneous fat-derived ASCs (scASCs), which demonstrated long-term propagation in culture under normoxic conditions with no evidence of expansion arrest. Comparisons of scASCs to aASCs demonstrated reduced ROS accumulation, markedly reduced cell death and reduced expression of both NOX1 and cytokines in scASCs.

ROS cellular levels are controlled by both ROS-generating enzymes and by ROS-quenching antioxidant enzymes.^31, 32^ The higher and stable expression of catalase and GSR in scASCs compared with aASCs from as early as passage 3 may indicate that the reduced ROS accumulation in scASCs arises from both a reduction in NOX1 activity and an increase in activity of antioxidant enzymes, such as catalase and GSR.

The distinct phenotypes of aASCs and scASCs were further demonstrated by the higher fat differentiation and migratory capabilities of scASCs. The limited fat differentiation potential of aASCs was shown to be IBMX dependent as, unlike scASCs, aASCs were able to differentiate to fat only in a differentiation medium containing IBMX. IBMX is known to increase intracellular cAMP, thus promoting protein kinase A (PKA) activation. PKA activation was previously demonstrated to inhibit NOX1 either through the downregulation of its RNA expression or through the phosphorylation of the NOX activator NOXA1 and its sequestration to the cytosol.^33, 34^ This implies that the weak fat differentiation potential of aASCs may be a consequence of NOX1-induced ROS accumulation. This was further substantiated by the ability of NOX1 inhibition to improve fat differentiation of aASCs. ROS accumulation to nontoxic levels is known to promote fat differentiation, although the exact source of ROS production remains controversial.^35^ Studies on 3T3L-1 adipocytes and MSCs indicate that both NOX and mitochondria are important sources for ROS accumulation during fat differentiation.^36, 37, 38, 39^ The reduced fat differentiation potential of aASCs probably occurs as a result of the chronic overaccumulation of ROS in these cells reaching toxic levels, as was indicated by their increased apoptosis. Such toxicity may hinder many physiological processes in the cells, including fat differentiation and the migration potential. Thus, NOX1 inhibition that prevented ROS overaccumulation was able to improve the differentiation and the migratory capacities of aASCs. Importantly, the NOX1 inhibitor does not block mitochondrial activity nor does it block the activity of other NOX family members, thereby allowing NOX1-independent ROS accumulation during fat differentiation.^22^ Thus, the distinct phenotypes that were displayed by aASCs and scASCs may arise from a tissue-specific signature.

### The tendency of aASCs to accumulate ROS through NOX activation may be explained by the perivascular origin of MSCs

Perivascular adipose tissue (PVAT) (adipocytes, fibroblasts, macrophages and stem cells progenitors T cells) was shown to express NOX and secrete ROS influencing hypertension and the metabolic syndrome.^40, 41, 42, 43^ The stromal vascular fraction (SVF) from which ASCs are derived is composed largely by PVAT.^44^ A perivascular origin had been suggested for MSCs.^2^ Adipose tissue is composed mainly of adipocytes, making vascular cells major contributors to the SVF from which ASCs are produced. Overexpression of p22phox (NOX membranal subunit) in vascular smooth muscle led to ROS overproduction and an increased tendency to develop signs of the metabolic syndrome in mice.^45^ NOX1 was also shown to be expressed in vascular smooth muscle cells, and its mRNA expression was upregulated and activated by vascular pathological stimuli, such as Ang II and PDGF.^46, 47, 48^ It is reasonable to speculate that the aASC signature stems from the physiological tendency of vascular cells within the fat abdominal tissue to overproduce ROS in an NOX-dependent manner. Further studies are needed to determine whether the unique phenotype demonstrated by aASCs is indeed related to the physiological role of their progenitors in fat tissue.

The current report demonstrates, for the first time, the induction of ROS accumulation by NADPH oxidase enzymes in primary cultured adipose-derived mesenchyme leading to their expansion arrest. ROS-induced expansion arrest was prevented by inhibition of ROS accumulation in two independent mechanisms, that is, by growing cells in low oxygen conditions as well as by growing the cells in the presence of an NOX1-specific inhibitor. Importantly, NOX-induced expansion arrest was dependent on the tissue origin of ASCs and occurred in cells that were derived from abdominal fat but not from subcutaneous fat. This points to a tissue-specific signature that affects the cells during their culture propagation. ROS are known to cause DNA damage and to promote genetic instability of cells. The results presented in the current study provide novel insights into the mechanisms that control ROS accumulation in ASCs. This may allow the development of safer expansion modalities of these cells.

## Materials and Methods

### Cell culture

MSCs were derived from the adipose tissues of Lewis rats (purchased from the Harlan Laboratories, Jerusalem, Israel). Cells were isolated from intra-abdominal or subcutaneous fat tissue using 0.1% collagenase (Sigma, St. Louis, MO, USA) and separated from fat by centrifugation. The cells were cultured in high glucose DMEM (Gibco, Paisley, Scotland, UK) supplemented with 10% FCS (Thermo Scientific HyClone, Tauranaga, New Zealand), 60 *μ*g/ml penicillin, 100 *μ*g/ml streptomycin, 50 *μ*g/ml kanamycin, sodium pyruvate (1 mM), l-glutamine (2 mM) and non-essential amino acids under atmospheric oxygen conditions. Media were changed two times a week, and cells were passaged once they had reached a confluence state.

### Hypoxia

Cells were cultured under the same conditions as indicated above; however, their culture was performed under 3% oxygen in a specialized Forma incubator (Thermo Scientific, Waltham, MA, USA) throughout the culture. The Tel Aviv Medical Center Institutional Animal Care and Use Committee approved all animal experiments.

### Inhibition of NOX1 activity with a specific inhibitor

Abdominal ASCs starting at passage 2 were cultured under normoxic conditions in the presence of a specific NOX1 inhibitor termed ML171 (Calbiochem, Darmstadt, Germany)^22^ in various concentrations: 0, 0.5, 2.5 and 5 *μ*M.

### Differentiation

#### Adipogenic differentiation

Confluent cells were cultured in adipogenic medium containing 10 *μ*g/ml insulin, 1 × 10^−6  ^M dexamethasone, 0.5 mM IBMX and 50 μM indomethacin (all from Sigma). After 21 days, the cells were fixed by 4% formalin (20 min at room temperature (RT)) and stained with 0.5% Oil Red (10 min, at RT) (Sigma). Following the staining, cells were photographed (Olympus IX71 microscope (Olympus, Tokyo, Japan) with a DP73 camera) and Oil Red O was extracted by 4% IGEPAL (Sigma) in isopropanol and quantified at 520 nm using a TECAN Infinite M200 plate reader (TECAN, Männedorf, Switzerland).

#### Osteogenic differentiation

Confluent cells were cultured in StemPro Osteogenesis Differentiation Kit (Gibco). After 21 days, the cells were fixed with 4% formalin (20 min at RT) and stained with 2% Alizarin Red (Sigma), pH 4.2 (10 min at RT). The induction medium was replaced every 3–4 days in all cases. Photographs were taken using an Olympus IX71 microscope with a DP73 camera.

### Flow cytometry

#### Surface marker analysis

Cells were harvested and incubated with a 4-color panel containing CD90.1-pacific blue and CD29-APC (Miltenyi Biotec, Auburn, CA, USA), CD45-APC/cy7 (BioLegend, San Diego, CA, USA) and live/dead-aqua blue dye (Invitrogen Molecular Probes, Eugene, OR, USA) for 30 min.

#### Cell cycle analysis

Cells were fixed with 70% ethanol/PBS, treated with RNaseA 0.4 mg/ml (Sigma) and stained with propidium iodide (PI) 0.1 mg/ml (Sigma).

#### Annexin/PI analysis

Cells were stained with an Annexin-APC/PI Detection Kit (BioLegend). Labeled cells were analyzed using a BD FACS Canto II flow cytometer (Becton Dickinson, San Jose, CA, USA). Data analysis was performed using the FlowJo software (Tree Star, Ashland, OR, USA).

### Real-time PCR

RNA was collected from subcutaneous or intra-abdominal ASCs at passages 2–5 using a Total RNA Mini Kit (Sigma). cDNA was prepared using M-MLV Reverse Transcriptase (Quanta Biosciences, Gaithersburg, MD, USA) according to the manufacturer's protocols. Real-time PCR was carried out using perfeCTa SYBR mix (Quanta Biosciences) and processed using Step One Plus (Applied Biosystems, Foster City, CA, USA) with normalization to Rn18 s. All the primers that were used in the study are summarized at Supplementary Table 1.

### ROS measurements

#### FACS analysis

Cells were trypsinized and incubated in PBS medium containing 10 *μ*M DCFDA (Molecular Probes, Carlsbad, CA, USA) for 30 min at 37 °C in the dark. ROS were detected by flow cytometry in a BD FACS Canto II flow cytometer (Becton, Dickinson, Franklin Lakes, NJ, USA), and data were analyzed using the FlowJo software (Tree Star).

#### Fluorescence microscopy

Cells were washed in PBS, incubated for 30 min in PBS containing 10 *μ*M DCFDA (Sigma) at 37 °C in the dark, washed two times in PBS and analyzed by Olympus I × 81-ZDC fluorescence microscope (Olympus). Fluorescence was determined on triplicate samples for each experiment.

### Senescence-associated β-galactosidase staining

ASCs (1 × 10^4^ or 2 × 10^4^) were seeded in a 6-well plate (Falcon, Franklin Lakes, NJ, USA), and pH-dependent senescence-associated β-galactosidase (SA-β-gal) activity was analyzed using the SA-β-gal Staining Kit (BioVision, Milpitas, CA, USA) when they had reached 80% confluence.

### Immunoblot analysis

For immunoblotting, proteins were separated by a SDS–polyacrylamide gel electrophoresis (PAGE), transferred to a nitrocellulose membrane and detected with the anti-NOX1 primary antibody (Sigma, Rehovot, Israel) and horseradish peroxidase-conjugated secondary antibody using enhanced chemiluminescence western blotting reagents (Thermo Scientific) and film (Fujifilm, Tokyo, Japan).

### Doubling time

ASCs (2 × 10^4^) were seeded in 60-mm plates (Falcon), collected upon reaching ~70% confluence and counted. Doubling time was calculated using the following formula: Td=(*t*2−*t*1) × log(2)/log(*q*2/*q*1), where (*t*2–*t*1) equals the time of incubation, (*q*1) is the initial amount of cells and (*q*2) is the final amount of the cells.

### Caspase activity assay

Caspase-3/7 activity was determined using Caspase-Glo 3/7 Assay Kit (Promega, Madison, WI, USA ). Briefly, the cells were trypsinized, washed, resuspended in growth medium and put into white 96-well plates at 10^4^ cells per well in 100 *μ*l. Equal volume of the caspase-glo 3/7 reagent was added, and the reaction mixtures were incubated for 40 min at room temperature. The intensity of luminescence was measured using a TECAN Infinite M200 plate reader (TECAN, Männedorf, Switzerland). As a positive control, scASCS were incubated with different concentration of H_2_O_2_ (0.2, 0.4 and 0.8 mM) for 12 h at 37 °C followed by caspase-3/7 activity evaluation.

### Scratch assay

Cells were grown to full confluence in six-well plates. Three parallel wounds were made on the cell monolayer using a sterile 1000 *μ*l pipette tip. Cells were then washed in PBS to remove debris and incubated for another 24 and 48 h in complete medium. Wound closure was photographed immediately, 24 and 48 h postwounding at the same site with an IX2-ZDC inverted microscope (Olympus). The extent of healing was defined by measuring the wounded area using CellSens Entry 1.3 software (Olympus) and reported as the percentage of wound healing with the following equation: %wound healing=(1−(wound area at *t*24 h (or 48 h)/wound area at *t*0)) × 100, where *t*0 is the time immediately after wounding.

### Statistical analysis

The statistical significance was determined using a two-tailed Student's *t*-test or Anova. *P*-values <0.05 was considered significant.

## Abbreviations

ASCs: adipose derived MSCs
aASCs: abdominal ASCs
scASCs: subcutaneous ASCs
MSCs: mesenchymal stem cells
ROS: reactive oxygen spices
