# Supplementary material for: NOX1-induced accumulation of reactive oxygen species in abdominal fat-derived mesenchymal stromal cells impinges on long-term proliferation
Source: Cell Death Dis. 2015 Apr 16;6(4):e1728–. doi: 10.1038/cddis.2015.84 (PMC4650551; doi:10.1038/cddis.2015.84)
Supplement: Supplementary Table 1 [file cddis201584x1.docx]

**Table 1: Real-time primers**

| **Gene name** | **Foword primer** | **Reverse primer** |
| --- | --- | --- |
| **NOX1** | 5'- CGGGGTCAAACAGAAGAGAG -3' | 5'- TGAGGACTCCTGCAACTCCT -3' |
| **SOD1** | 5'- GCCAATGTGTCCATTGAAGA-3' | 5'- CACCTTTGCCCAAGTCATCT-3' |
| **SOD2** | 5'- GGCCAAGGGAGATGTTACAA-3' | 5'- TAGGGCTCAGGTTTGTCCAG-3' |
| **Prdx3** | 5'- GCGCTCAGAGGTCTCTTCAT-3' | 5'- CTGGAACGCCTTTACCAAAC-3' |
| **Catalase** | 5'- ACCAGGGCATCAAAAACTTG-3' | 5'- AGGATGGGTAATTGCCACTG-3' |
| **Gpx3** | 5'- CCATGAAGATCCATGACATCC-3' | 5'- AGGATGTCCATCTTGACGTTG-3' |
| **Gpx4** | 5'- TAAGTACAGGGGTTGCGTGTG-3' | 5'- GTAAACCACACTCGGCGTATC-3**'** |
| **GSR** | 5'- GGGGATTCAGACTGATGACAA-3' | 5'- AGAAGTGCTTTCCCACAGACA -3' |
| **IL-6** | 5'- ACCACCCACAACAGACCAGT-3' | 5' –CAGAATTGCCATTGCACAAC – 3' |
| **CXCL-1** | 5'- GCACCCAAACCGAAGTCATA -3' | 5' – GGGGACACCCTTTAGCATCT – 3' |
| **Adiponectin** | 5'-TGTCTGTACGAGTGCCAGTG-3' | 5' –CCTTCATGACTGGGCAGGATT– 3' |
| **Rn18s** | 5'-CGAAAGCATTTGCCAAGAAT-3' | 5'-AGTCGGCATCGTTTATGGTC-3' |
