# Supplementary figures and images for: NOX1-induced accumulation of reactive oxygen species in abdominal fat-derived mesenchymal stromal cells impinges on long-term proliferation
Source: Cell Death Dis. 2015 Apr 16;6(4):e1728–. doi: 10.1038/cddis.2015.84 (PMC4650551; doi:10.1038/cddis.2015.84)

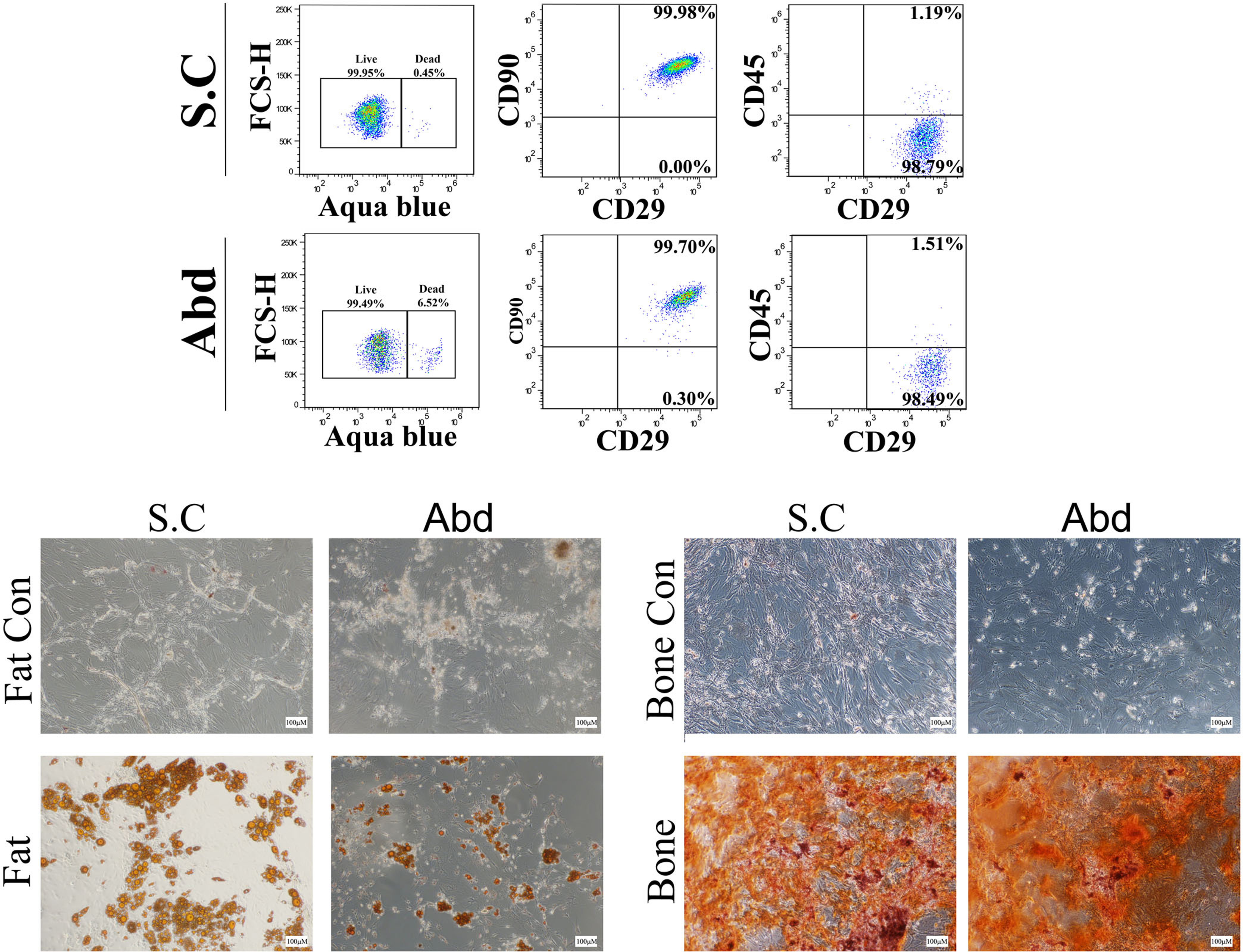

Supplement: Supplementary Figure 1 [file cddis201584x2.tif]

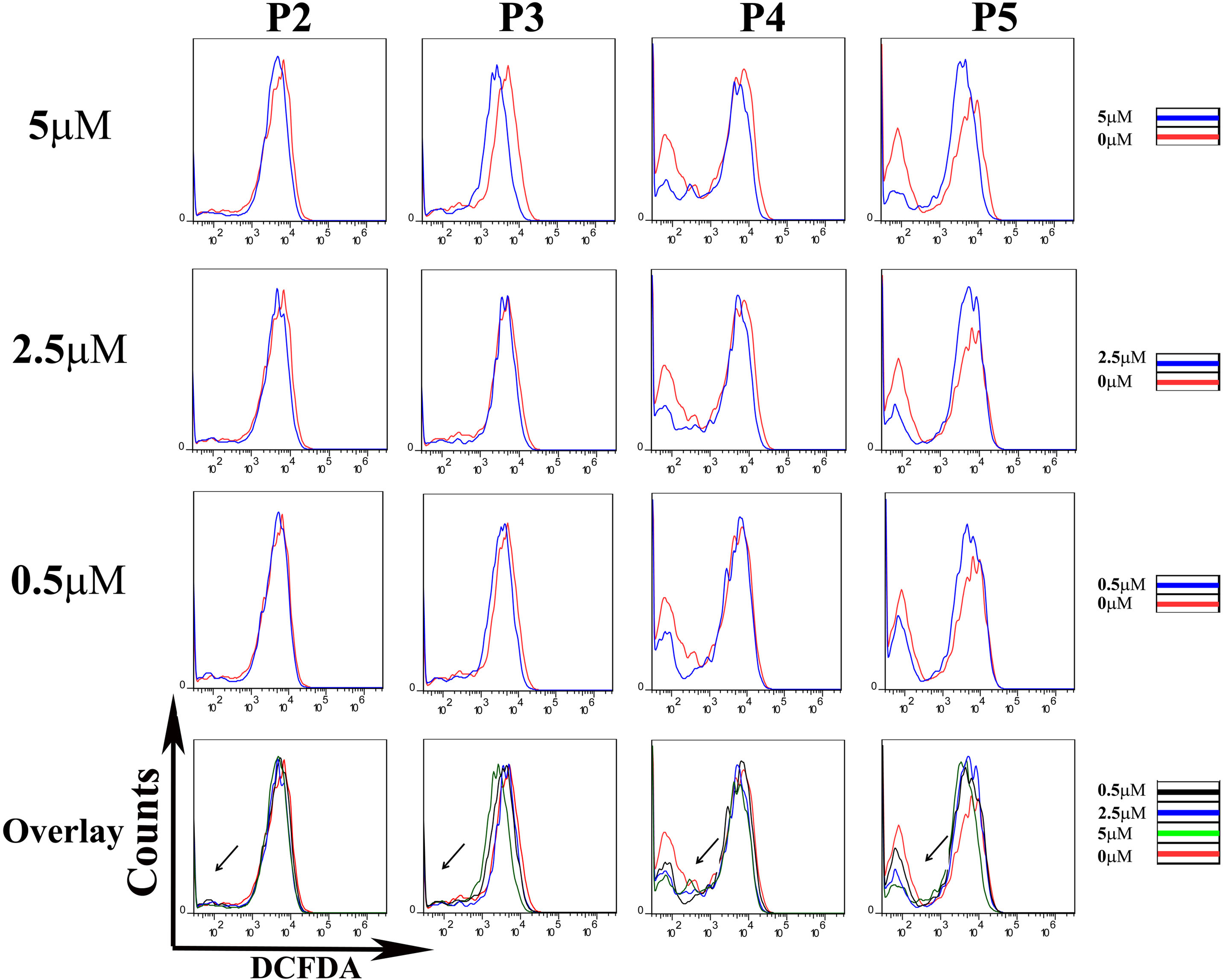

Supplement: Supplementary Figure 2 [file cddis201584x3.tif]

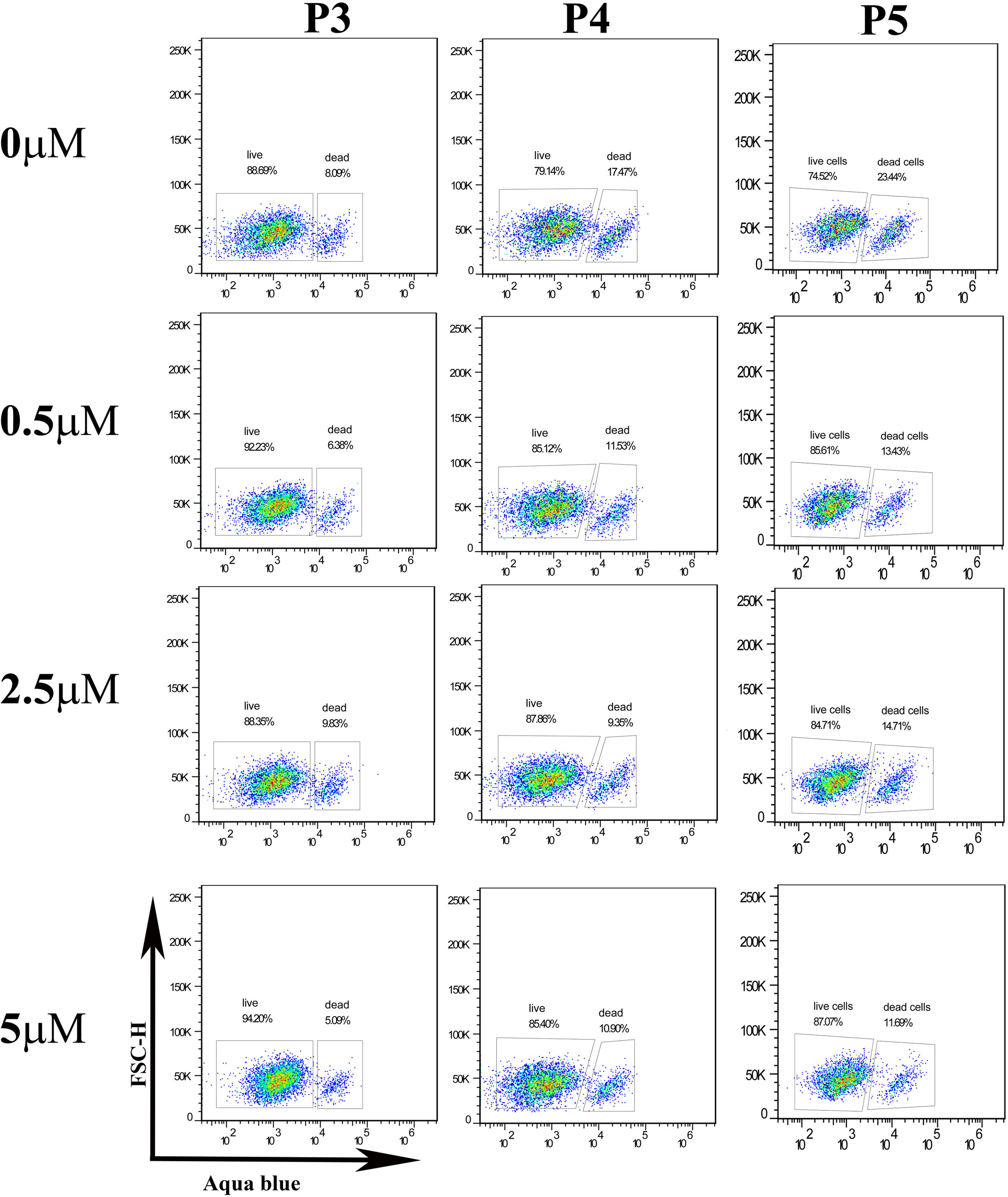

Supplement: Supplementary Figure 3 [file cddis201584x4.tif]

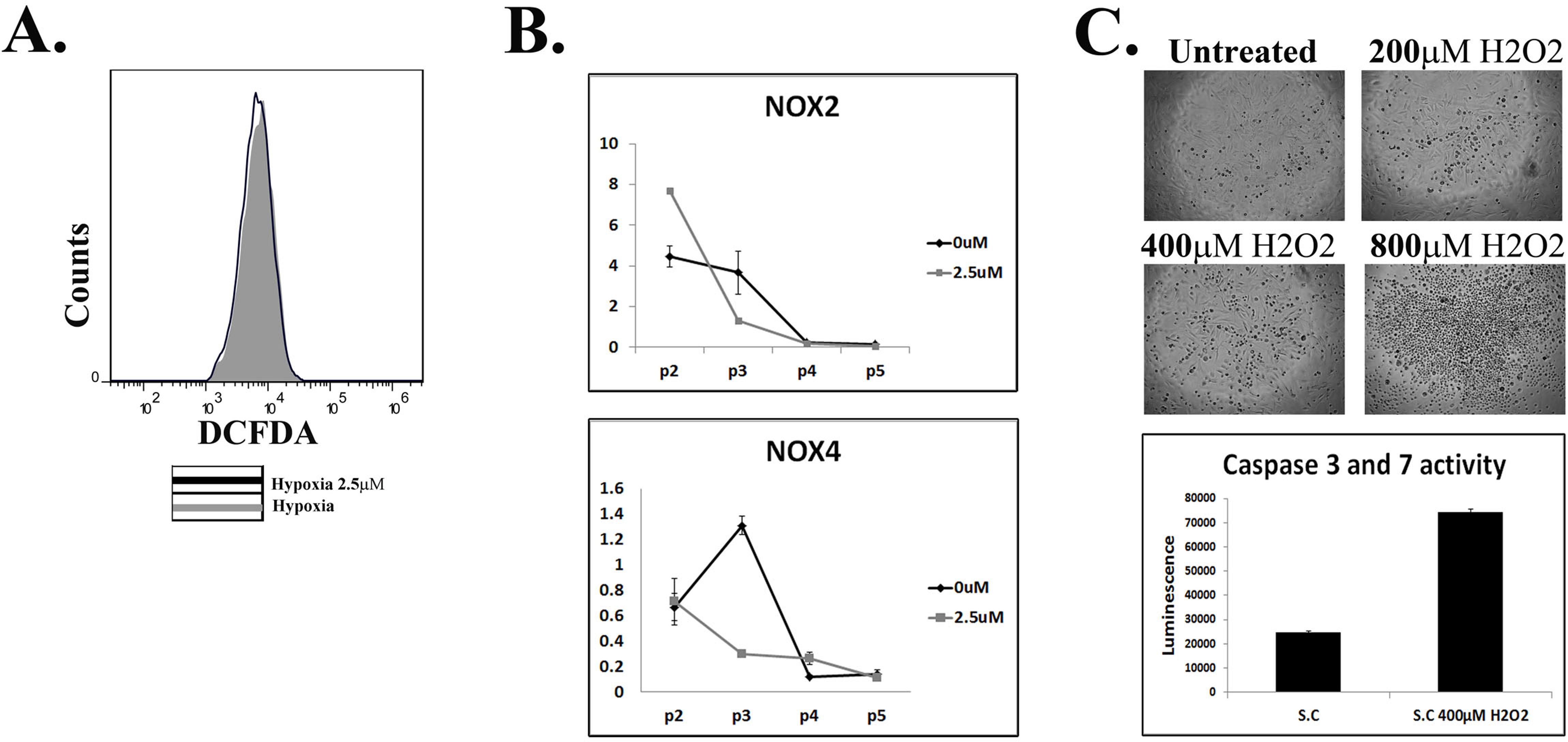

Supplement: Supplementary Figure 4 [file cddis201584x5.tif]
