# Supplementary material for: NOX1-induced accumulation of reactive oxygen species in abdominal fat-derived mesenchymal stromal cells impinges on long-term proliferation
Source: Cell Death Dis. 2015 Apr 16;6(4):e1728–. doi: 10.1038/cddis.2015.84 (PMC4650551; doi:10.1038/cddis.2015.84)
Supplement: Supplementary Information [file cddis201584x6.doc]

#### Supplementary Figure 1. Characterization of rat subcutaneous (S.C) and aASCs (Abd). (A) A four-color FACS panel used for ASCs characterization. Cells were stained with αCD29, αCD90 and αCD45 antibodies and aqua blue dye for live and dead staining. Cells were then subjected to a 4-color flow cytometry analysis. Each graph represents staining with the indicated antibody against a surface marker using unstained cells as control. Only live cells stained, (aqua blue negative cells) were evaluated for their marker staining. (B) Cells were cultured with or without induction media for 3 weeks to induce cell differentiation. Differentiation into bone and fat was detected by Alizarin red and Oil red O staining, respectively.

**Supplementary Figure 2.** A specific NOX1 inhibitor (ML171) reduces ROS accumulation in a dose-dependent manner. Abdominal ASCs were cultured under normoxic oxygen conditions (21% oxygen) and treated with increasing concentrations of the NOX1-specific inhibitor ML171 (0, 0.5, 2.5 and 5µM). ROS accumulation was detected by FACS analysis using DCFDA staining. The population with a low DCFDA signal (arrow) was verified to represent aqua blue dead cells (data not shown).

**Supplementary Figure 3.** Specific NOX1 inhibitor (ML171) reduces cell death. aASCs (Abd) were cultured under normoxic oxygen conditions (21% oxygen) and treated with increasing concentrations of a NOX1-specific inhibitor (0, 0.5, 2.5 and 5µM). Cells were stained by aqua blue dye (an amine viability dye) and analyzed by FACS. Aqua blue-positive cells represent the dead cells and the negative aqua blue cells represent the live cells. Results show increased dead cells in untreated (0µM) aASCs compared to aASCs treated with indicated concentrations of NOX1inhibitor.

**Supplementary Figure 4.** (A) aASCs were grown under hyopoxic conditions with (2.5µM) or without a specific NOX1 inhibitor. ROS accumulation was evaluated by DCFDA staining and analysis of the cells by FACS. (B) Abdominal ASCs were propagated under normoxic oxygen conditions (21% oxygen) in the presence of a NOX1-specific inhibitor ML171(2.5µM) and the RNA expression of the indicated genes were evaluated using qRT-PCR. (C) S.C. cells were incubated with increasing concentrations of H_2_O_2_ (0.2, 0.4 and 0.8 mM) for 12h at 37^o^C followed by imaging and by caspase 3/7 activity evaluation.

**Supplementary Table 1.** A list of the primers that were used in the current study.
